# Effects of aerobic and resistance training on walking and balance abilities in older adults with Parkinson’s disease: A systematic review and meta-analysis

**DOI:** 10.1371/journal.pone.0314539

**Published:** 2025-01-09

**Authors:** Huishan Song, Sheng Ge, Ji Li, Chendao Jiao, Linghua Ran

**Affiliations:** 1 School of Sports Science, Harbin Normal University, Harbin, China; 2 Central Hospital of Heilongjiang Provincial Prison Administration, Harbin, China; University of Catania, ITALY

## Abstract

**Objective:**

To explore the impact of aerobic and resistance training on walking and balance abilities (UPDRS-III, Gait Velocity, Mini-BESTest, and TUG) in individuals with Parkinson’s disease (PD).

**Method:**

All articles published between the year of inception and July 2024 were obtained from PubMed, Embase, and Web of Science. Meta-analysis was conducted with RevMan 5.4.

**Result:**

Research from 15 randomized controlled trials, comprising 792 older patients with a diagnosis of PD, was included in the analysis. Aerobic training (AT) and Resistance training (RT) significantly improved UPDRS-III (AT, SMD = -5.69, 95% CI: -8.67 to -2.71, *p* = 0.0002, I^2^ = 82%; RT, SMD = -3.01, 95% CI: -4.89 to -1.12, p = 0.002, I^2^ = 0%) and Gait Velocity(AT, SMD = 0.88, 95% CI: 0.58 to 1.18, *p* < 0.00001, I^2^ = 42%; RT, SMD = 0.52, 95% CI: 0.10 to 0.94, *p* = 0.01, I^2^ = 55%). AT showed no difference in Mini-BESTest (AT, SMD = 2.12, 95% CI: -0.70 to 4.94, *p* = 0.14, I^2^ = 90%). RT showed no change in TUG (RT, SMD = -0.03, 95% CI: -1.60 to 1.54, p = 0.97, I2 = 63%).

**Conclusions:**

Resistance training stood out as the most effective approach to boost performance on the mini-BESTest. Conversely, aerobic exercise proved to be superior for improving the UPDRS-III, gait velocity and TUG scores.

## 1. Introduction

Parkinson’s disease (PD) is a neurological condition marked by the gradual decline of dopamine-producing neurons in the substantia nigra striatum [1]. Clinical manifestations include resting tremor, bradykinesia, muscle tonus, and postural abnormalities. The estimated prevalence of PD in individuals aged over 60 is 1% [2]. Gait abnormality is the main clinical manifestation of PD, which is primarily categorized into early, middle and late stages [3]. Early gait is characterized by a decrease in step length, stride length and stride speed, an increase in gait variability, a decrease in the swing of the hands, a prolongation of the support phase of the lower limbs, and a change in the coordination of bilateral movements, and other minor manifestations, which are then followed by the development of a panicked gait and a freezing gait in the middle and late stages [4, 5]. The compromised functioning of both central and peripheral nerves results in weakened muscle strength [6]. This decline subsequently impacts balance and creates an unsteady gait, ultimately hindering the individual’s mobility. As a consequence, the patient becomes more vulnerable to falls, significantly diminishing their overall quality of life [7].

Individuals with PD require both short and long term rehabilitation strategies that are sustainable to restore their functioning and improve their ability to engage in everyday activities [8]. At this stage, the majority of patients receive levodopa or alternative anti-PD therapies, but the efficacy in terms of walking ability and balance function among patients is unsatisfactory [9, 10]. As a nonpharmacological intervention, exercise has been confirmed by clinical trials to produce significant physiological benefits and to prevent and delay the development of Parkinson’s disease [11–14]. Currently, an increasing number of researches are focusing on how exercise impacts motor function, walking and balance ability among older individuals with PD [15–17]. At present, there is a notable lack of research exploring the impacts of aerobic exercise and strength training on the walking and balance skills of individuals with Parkinson’s disease. Therefore, it is essential to conduct a systematic review and meta-analysis of such studies to summarize and evaluate clinical trial data for the clinical application of exercise in PD.

## 2. Materials and methods

### 2.1 Search strategy

This systematic review adhered to the PRISMA guidelines for reporting systematic reviews and meta-analyses (S1 File). It was also pre-registered in the international database of prospectively registered systematic reviews in health and social care (PROSPERO: CRD42024557922) [18].

We conducted a search across PubMed, Embase, and Web of Science, three separate electronic databases, to find relevant articles published between the year of inception and July 2024. The terms utilized for electronic searches included: (Aerobic OR resistance OR jogging OR HIIT OR physical activity OR walking OR MICT) AND (Parkinson’s disease OR idiopathic parkinsonism) AND (gait OR walking OR balance OR motor symptoms OR postural control) AND (randomized controlled trial). As an example, the specific search strategy for the PubMed database is described in S2 File. Additionally, we manually searched reference lists from pertinent systematic reviews to identify further eligible studies.

### 2.2 Selection criteria

The title and abstract of the article undergo scrutiny by two authors (HS Song and S GE), with a final evaluation and decision made by a third author (LH RAN).

Researches were deemed to be included if they satisfied these particular requirements: (1) satisfied the international diagnostic criteria for PD and had a definitive and stable diagnosis of the condition; (2) examined the impact of aerobic or resistance exercise; (3) included a comparator group made up of patients with PD receiving other forms of rehabilitative therapies; (4) assessed outcomes related to gait (using measures such as the freezing of gait questionnaire [FOG-Q] and the 6-meter walking test [6MWT]) and dynamic balance (evaluated through the timed up and go [TUG] test, Berg balance scale [BBS], Mini-balance evaluation systems test [MiniBESTest], and dynamic gait index [DGI]); (5) were designed as clinical randomized controlled trials (RCTs).

Research were deemed ineligible if they: (1) were repeated publications; (2) lacked a control group; (3) were subjects with atypical parkinsonism; (4) omitted the entire text.

### 2.3 Data extraction

Information was gathered from the selected researches, which encompassed elements including the primary author’s name, publication year, the size of the sample, participant information (age, gender, numbers, Hoehn-Yahr staging), intervention design (both experimental and control groups), treatment specifics (frequency and duration), and outcome measures. Literature screening was done by 2 researchers (HS Song and J LI) alone and then the results of the 2 screening were compared. In case of disagreement a third researcher (LH RAN) decided on inclusion.

### 2.4. Risk of bias and quality assessment

We evaluated the potential for bias in each of the studies we included by utilizing the guidelines outlined in the Cochrane Handbook for Systematic Reviews of Interventions and applying the PEDro scale [19]. The quality assessment included the creation of randomized sequences, allocation concealment, blinding of investigators and subjects, blinding of study results, fullness of outcome data, selective presentation of trial outcomes and other biases. For every requirement that was satisfied, one point was awarded. The evaluation scale extended from 0 to 10, where ratings of 9 to 10 signified outstanding quality, scores between 6 and 8 represented good quality, ratings of 4 to 5 indicated acceptable quality, and any score below 4 denoted subpar quality [20]. Moreover, due to the difficulties in estimating key data, the missing data was handled by excluding studies from the meta-analysis. Additionally, risk was assessed according to the criteria: unclear risk, low risk, and high risk. Two researchers (HS Song and CD JIAO) independently evaluated the quality of the literature and then cross-checked, with a third researcher (LH RAN) determining the risk level for ambiguous literature.

### 2.5. Statistical analysis

The software RevMan 5.4 for Windows was utilized to conduct the meta-analyses. The standardized mean difference (SMD) was selected as a helpful indicator, and the variance was given a 95% confidence interval (CI). If there was no discernible difference according to the heterogeneity test, the fixed-effects model was applied. Otherwise, the random-effects model was used. Heterogeneity was evaluated using the I^2^ statistic. When the I^2^ was greater than 75%, heterogeneity was considered high. In-depth subgroup analyses were performed according to various comparison types. Sensitivity analysis was used to assess the stability of the findings by excluding each study individually. Additionally, we evaluated publication bias by visually examining funnel plots and employing statistical methods, provided that a minimum of ten research projects were incorporated into the meta-analysis, in accordance with the guidelines set by the Cochrane Collaboration.

## 3. Results

### 3.1 Search and screening outcomes

**Fig 1:**
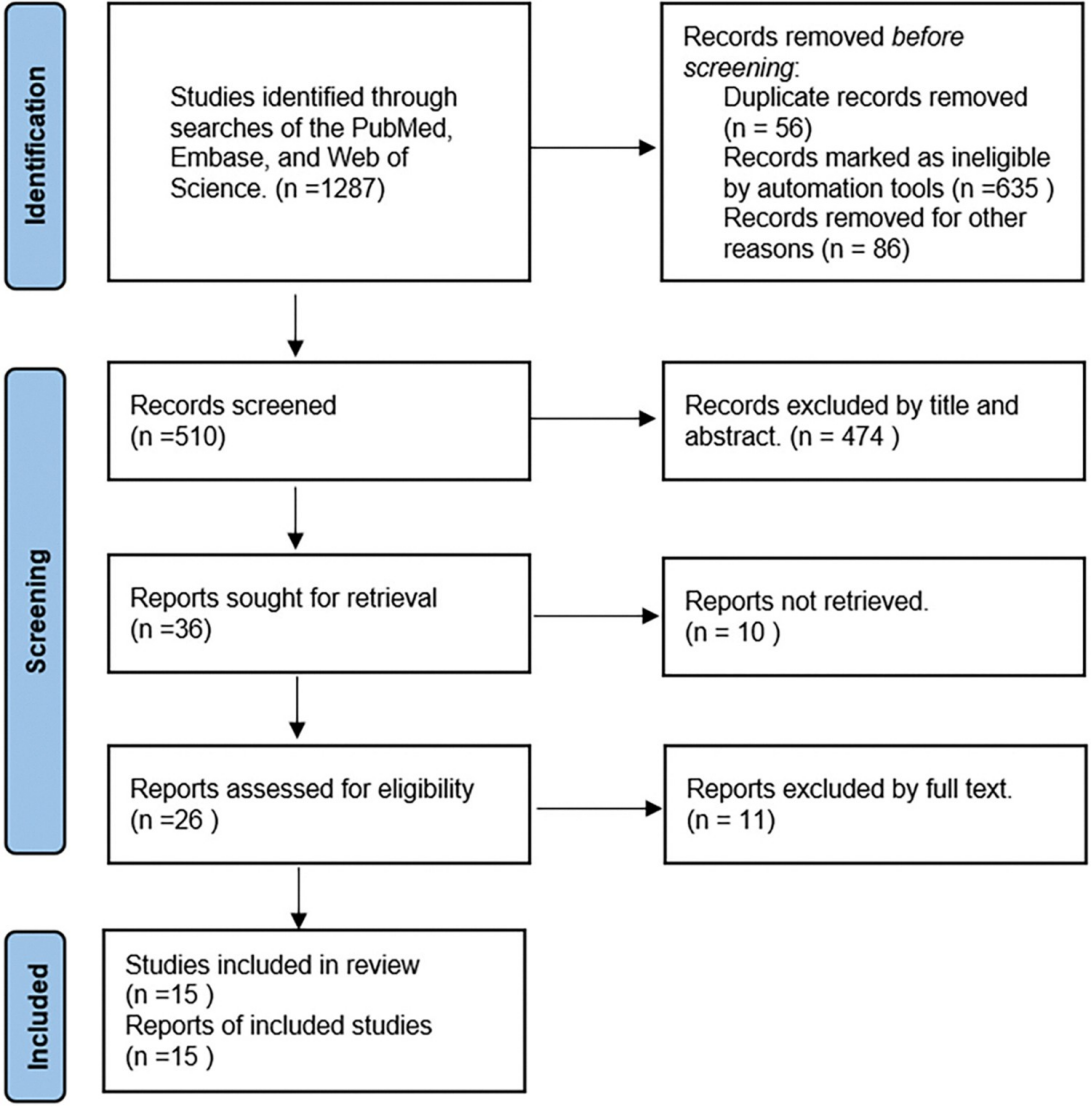
The flowchart based on PRISMA standards.

The selection process is outlined in Fig 1. Studies identified through searches of the PubMed, Embase, and Web of Science. A total of 1287 records were retrieved (S3 File). The standards for both inclusion and exclusion were then utilized for the research titles and abstracts through screening. This process led to the elimination of 1,251 studies that did not satisfy the inclusion requirements. From the remaining 36 studies, the full texts were examined to enforce the exclusion criteria. Lastly, this meta-analysis contained a total of 15 papers.

### 3.2 Research characteristics

S4 File outlines the characteristics of 15 researches, detailing publication dates, sample sizes, and the demographics of the populations examined by gender and age. Relevant data extraction information are summarized in S5 File. It also includes Hoehn-Yahr staging, the design of the treatment interventions concerning mode, intensity, duration, and frequency, along with information about the control groups and the outcome measures assessed [21–35]. This analysis encompassed findings from 15 randomized controlled trials involving a total of 792 older people with PD. Among these participants, 58.6% were male while 41.4% were female. The 15 studies were broadly categorized into aerobic and resistance exercise based on their exercise content. Exercise sessions varied in length from 30 to 60 minutes, and the duration of the exercises ranged from 7 to 24 weeks. Additionally, the frequency of the exercise interventions varied from 1 to 5 times per week. Measured outcomes consisted mainly of gait (FOG-Q, 6MWT) and dynamic balance (TUG, BBS, MiniBESTest, and DGI). The specifics of the researches are displayed in S4 File.

### 3.3 Study quality and publication bias

**Fig 2:**
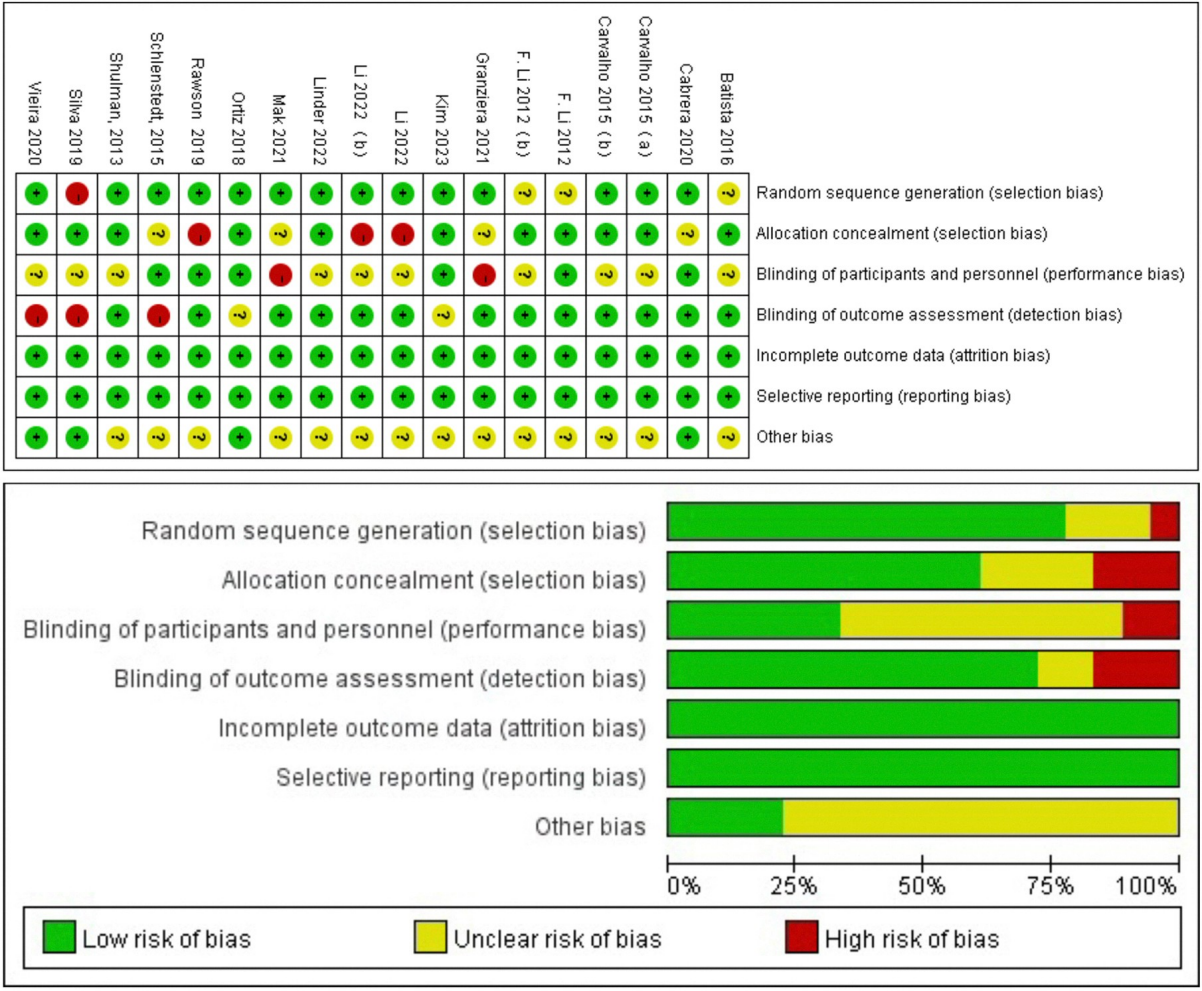
Evaluation of bias risk based on cochrane collaboration guidelines.

**Fig 3:**
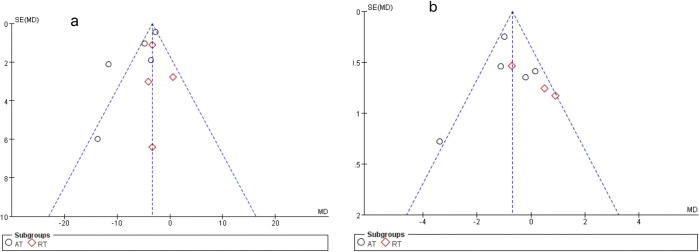
Funnel plot of UPDRS-III (3a) and TUG (3b).

The overall quality of the enrolled publications was found to be relatively high in the quality evaluation of RCTs. S6 File displays each study’s PEDro scores for the quality evaluation. Fig 2 and S7 File displays specifics regarding the included studies’ risk of bias. The PEDro scores of most studies were more than 6, and only one study had a score of 5. The assessment of bias showed that a relatively tiny percentage of studies were at high risk, with the majority falling into the "unclear risk of bias" category. There were no notable biases to the general results of the article. The risk assessment was then separately entered into Review Manager (Rev Man 5.4) for each trial, resulting in a risk of bias summary that was provided together with the meta-analysis findings. Because there are more researches in the UPDRS-III and stride TUG categories, we conducted a publication bias analysis on them. According to Egger’s test, there was no evidence of publication bias among all treatment research projects, which included the evaluations of UPDRS-III (Fig 3A) or TUG (Fig 3B).

### 3.4 Outcomes

#### 3.4.1 Motor section of UPDRS

**Fig 4:**
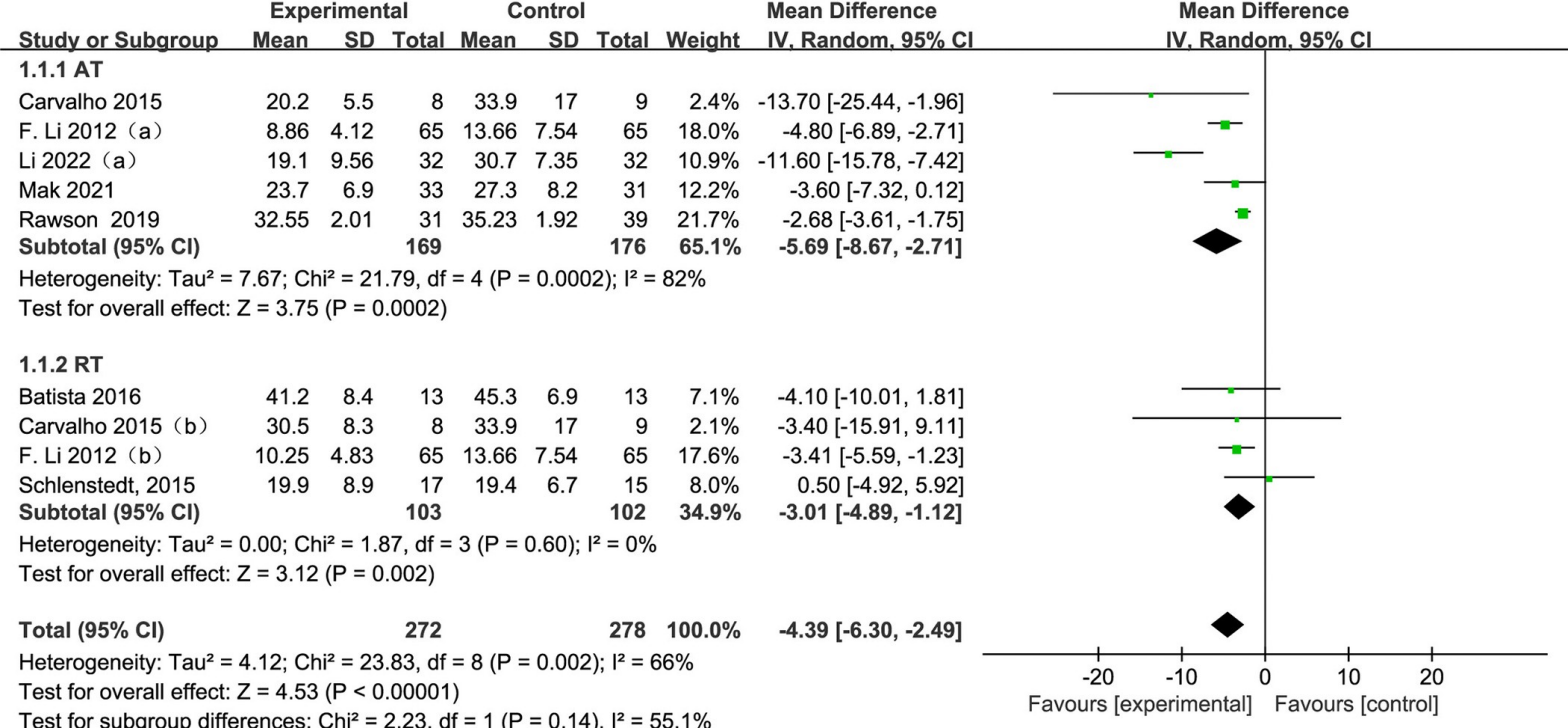
The forest plot for UPDRS-III comparison.

Fig 4 presents a forest plot of a subgroup analysis of 9 studies based on aerobic training and resistance training methods, using UPDRS-III as the outcome for PD. Among subgroup analysis of exercise methods, 5 subjects [22, 25, 26, 28, 30] used aerobic training and 4 subjects [22, 25, 31, 34] used resistance training. The exercise group’s UPDRS-III significantly decreased as compared to the control group in UPDRS-III when all exercise modes were combined (SMD = -4.39, 95% CI: -6.39 to -2.49, *p* < 0.00001, I^2^ = 66%). When conducting a subgroup analysis, UPDRS-III scores were significantly decrease in both AT and RT groups compared to the control group. Additionally, the effect of UPDRS-III with aerobic training is proved to be higher than resistance training group. (AT, SMD = -5.69, 95% CI: -8.67 to -2.71, *p* = 0.0002, I^2^ = 82%; RT, SMD = -3.01, 95% CI: -4.89 to -1.12, *p* = 0.002, I^2^ = 0%).

#### 3.4.2 Gait velocity

**Fig 5:**
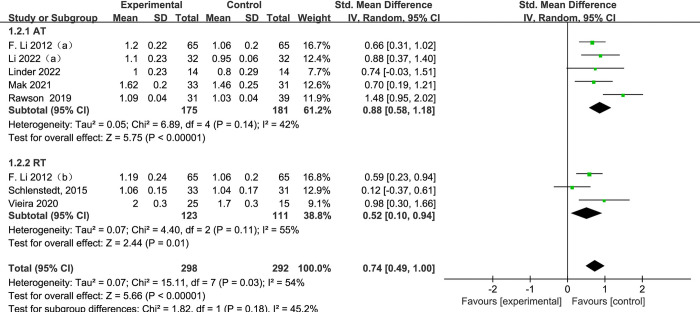
The forest plot for gait velocity comparison.

Fig 5 presents a forest plot of a subgroup analysis of 9 studies based on aerobic training and resistance training methods, using gait velocity as the outcome for PD. Among subgroup analysis of exercise methods, 5 subjects [25–28, 30] used aerobic training and 3 subjects [25, 31, 35] used resistance training. When contrasting the exercise group to the control group, the gait velocity of exercise group increased significantly (SMD = 0.74, 95% CI: 0.49 to 1.00, *p* <0.00001, I^2^ = 54%). When conducting a subgroup analysis, gait velocity were significantly increasing in both AT and RT groups compared to the control group, and the effect of gait velocity with aerobic training is proved to be higher than resistance training group. (AT, SMD = 0.88, 95% CI: 0.58 to 1.18, *p* < 0.00001, I^2^ = 42%; RT, SMD = 0.52, 95% CI: 0.10 to 0.94, *p* = 0.01, I^2^ = 55%).

#### 3.4.3 Mini-BESTest

**Fig 6:**
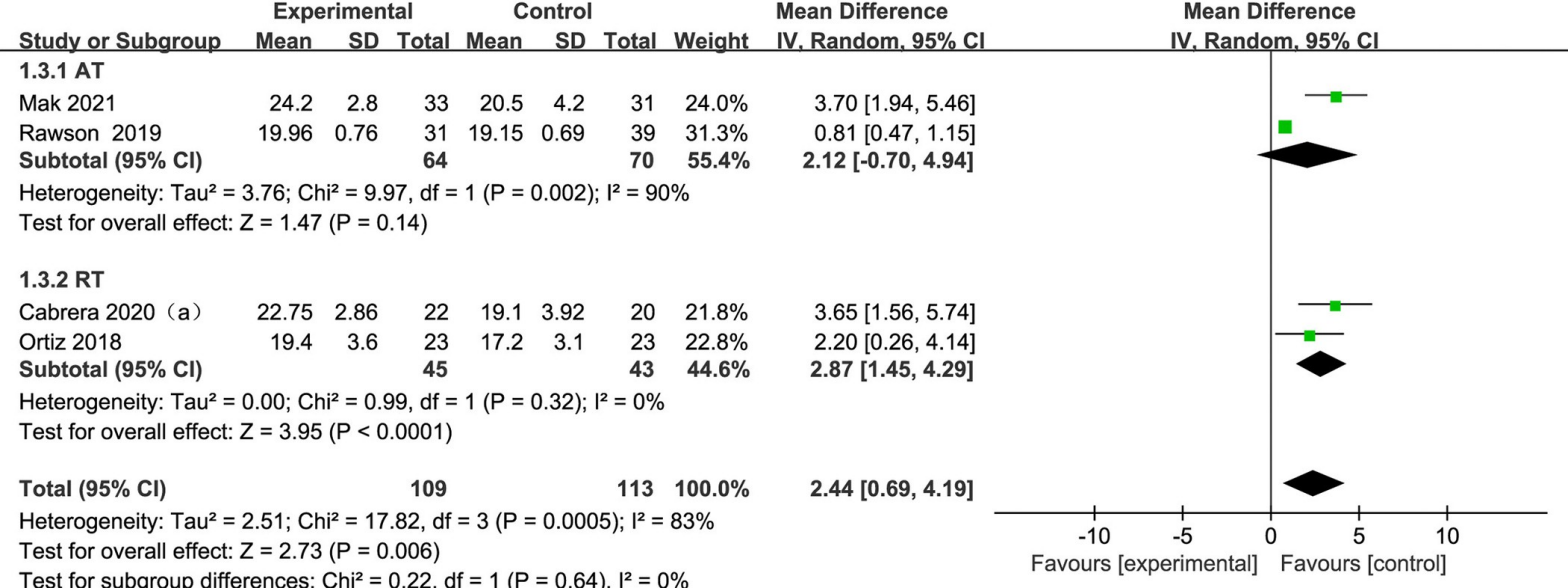
The forest plot for Mini-BESTest comparison.

Fig 6 presents a forest plot of a subgroup analysis of 4 studies based on aerobic training and resistance training methods, using Mini-BESTest as the outcome for PD. Among subgroup analysis of exercise methods, 2 subjects [28, 30] used aerobic training and 2 subjects [21, 29] used resistance training. When contrasting the exercise group to the control group, the Mini-BESTest of exercise group increased significantly (SMD = 2.44, 95% CI: 0.69 to 4.19, *p* < 0.05, I^2^ = 83%). When conducting a subgroup analysis, the resistance training group demonstrated a notable improvement of Mini-BESTest relative to the control group. (RT, SMD = 2.87, 95% CI: 1.45 to 4.29, *p* < 0.0001, I^2^ = 0%). There is no notable diversity between the aerobic training group and the control group (AT, SMD = 2.12, 95% CI: -0.70 to 4.94, *p* = 0.14, I^2^ = 90%).

#### 3.4.4 Timed-up-and-go test

**Fig 7:**
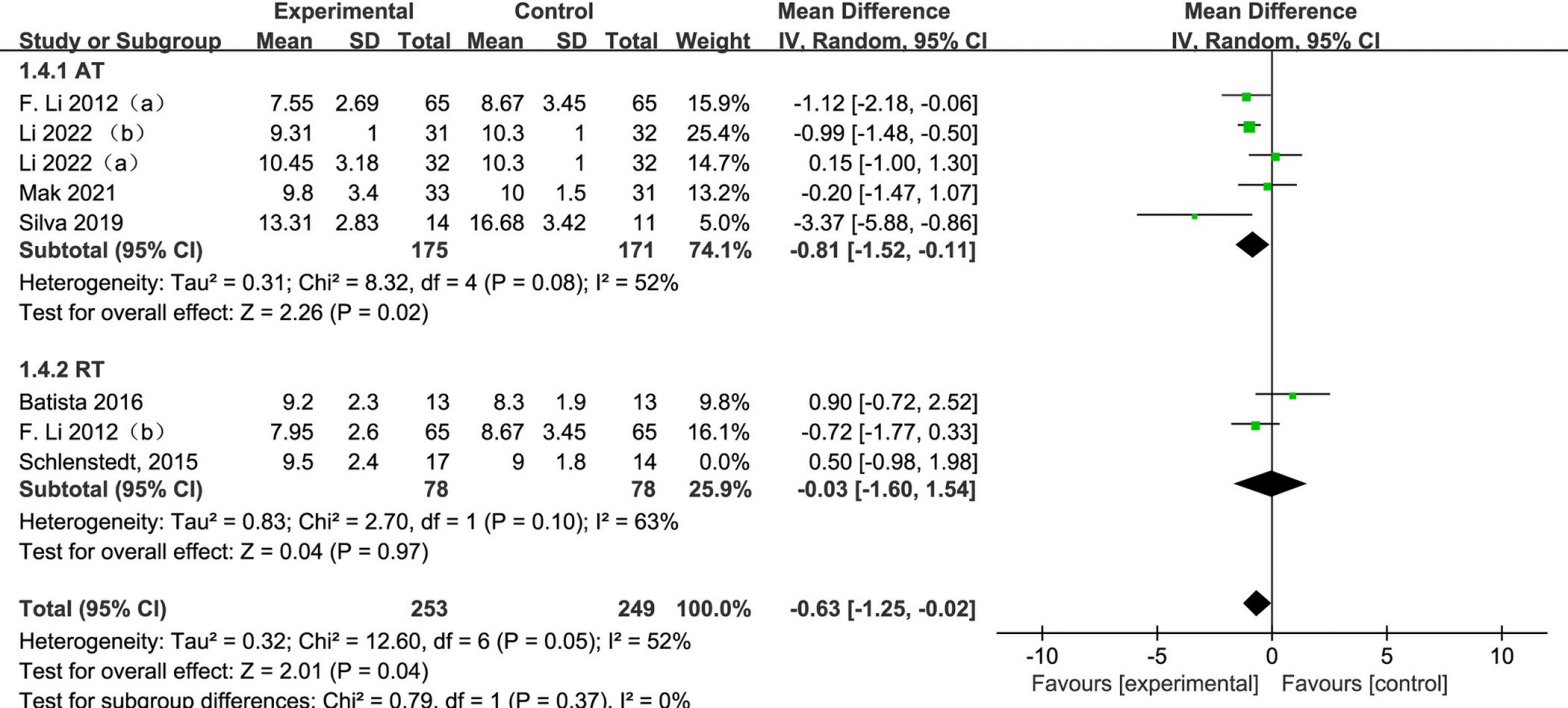
The forest plot for TUG comparison.

Fig 7 presents a forest plot of a subgroup analysis of 8 studies based on aerobic training and resistance training methods, using TUG as the outcome for PD. Among subgroup analysis of training modes, 5 subjects [25, 26, 28, 33] used aerobic training and 3 subjects [25, 31, 34] used resistance training. When contrasting the exercise group to the control group, the TUG text of exercise group decreased significantly (SMD = -0.63, 95% CI: -1.25 to -0.02, *p* = 0.04, I^2^ = 52%). When conducting a subgroup analysis, in comparison to the control group, the TUG of the aerobic training group significantly declined (AT, SMD = -0.81, 95% CI: -1.52 to -0.11, *p* < 0.08, I^2^ = 52%). There is no significant diversity between the resistance training group and the control group (RT, SMD = -0.03, 95% CI: -1.60 to 1.54, *p* = 0.97, I^2^ = 63%).

## 4. Discussion

This study conducted a systematic review and meta-analysis of 15 researches to compare the influence of aerobic training and resistance training on gait and balance abilities in patients with PD. Research findings indicated that resistance training stood out as the most effective approach to boost both gait speed and performance on the mini-BESTest. Conversely, aerobic exercise proved to be superior for improving the UPDRS-III and TUG scores. As findings in this research, not each exercise mode could improve all sides of walking and balance performance in individuals with PD. Therefore, these outcomes highlighted the necessity of recognizing training modalities that enhance gait and balance in older people with PD.

The benefits of exercise training on the motor symptoms among PD patients were widely recognized [36]. Exercise as an adjunctive and complementary therapy could improve corpus striatum plasticity and increase dopamine release. Studies [37, 38] had shown that exercise training could reduce the risk of deterioration of PD nerve function deficit. As a complementary and alternative treatment, exercise could help with motor and non-motor disorders in PD patients. As a beneficial intervention method to alleviate PD motor symptoms, aerobic exercise had been proved to have neuroprotective benefits in recent years [39]. Sung YH [40] found that aerobic exercise could improve the motor dysfunction of PD mice by inhibiting Erk/MAPK signaling pathway upstream of the striatum. Resistance training outperforms aerobic exercise in enhancing muscle strength, endurance, and muscle mass. Zhu PA [41] reached a conclusion that resistance training could promote the elastic function of muscles, enhanced the stability and coordination of lower limbs, and improved the gait and balance function of patients. The study offers important insights for individuals focused on developing health strategies and exercise rehabilitation programs for patients with PD, with the goal of slowing down the progression of motor decline and improving their motor abilities.

The study also had some limitations. There was a high risk of selection bias in four of the trials. The quality concerns surrounding these studies could jeopardize the validity of our research. While we made adjustments for the heterogeneity among the studies when integrating their original findings, we remained unable to fully account for the variations present. In addition, due to methodological limitations of meta-analysis, the distribution of covariates within subgroups was not uniform during the subgroup analysis, which may lead to some deviation in the final results. In the future, the objective criteria should be used to evaluate the indicators, minimize the risk of bias, and formulate effective exercise programs.

## 5. Conclusions

The findings show that both aerobic and resistance training have positive effects on UPDRS-III and gait velocity. Moreover, for UPDRS-III, gait velocity and TUG in PD patients, aerobic exercise intervention has a significant effect. For Mini-BESTest in patients, the effect of resistance exercise was significant. Overall, exercise have positive effects on walking and balance abilities among older adults with PD. However, the optimal exercise intervention program for Parkinson’s disease has yet to be determined. Therefore, it is crucial to carry out further study for maximizing the efficacy of exercise intervention programs.

## Supporting information

S1 File PRISMA checklist. (DOCX)

S2 File Search strategy. (DOCX)

S3 File List of all articles. (XLSX)

S4 File Included studies. (DOCX)

S5 File Data extracted from the primary research sources. (DOCX)

S6 File Quality evaluation. (DOCX)

S7 File Risk of bias quality assessment checklist. (XLSX)
