# Supplementary material for: Effects of aerobic and resistance training on walking and balance abilities in older adults with Parkinson’s disease: A systematic review and meta-analysis
Source: PLoS One. 2025 Jan 9;20(1):e0314539. doi: 10.1371/journal.pone.0314539 (PMC11717240; doi:10.1371/journal.pone.0314539)
Supplement: S2 File — (DOCX) [file pone.0314539.s002.docx]

S2 File. Search strategy

|  | **Search term** |
| --- | --- |
| #1 | (Parkinson Disease [MeSH Terms]) OR ( Idiopathic Parkinson's Disease [Title/Abstract] OR Lewy Body Parkinson's Disease [Title/Abstract] OR Primary Parkinsonism [Title/Abstract] OR Parkinson's Disease, Lewy Body [Title/Abstract] OR Parkinson Disease, Idiopathic [Title/Abstract] |
| #2 | (Aerobic training [MeSH Terms]) OR Aerobic Exercise [Title/Abstract] OR Aerobic Exercises [Title/Abstract] OR Aerobic Exercise Training [Title/Abstract] OR Exercise Trainings [Title/Abstract]) OR jogging [Title/Abstract] OR HIIT [Title/Abstract] OR MICT [Title/Abstract] OR walking [Title/Abstract] |
| #3 | (Resistance training [MeSH Terms]) OR Resistance Exercise [Title/Abstract] OR Resistance Exercises [Title/Abstract] OR Resistance Exercise Training [Title/Abstract] |
| #4 | #2 OR #3 |
| #5 | (Motor symptoms [MeSH Terms] OR postural control [Title/Abstract] OR Physical Performances [Title/Abstract] OR Gait [Title/Abstract] OR walking [Title/Abstract] OR balance [Title/Abstract]) |
| #6 | RCT[Title/Abstract] OR randomized controlled trial [Title/Abstract] OR randomized controlled trials [Title/Abstract] |
| #7 | #1 AND #4 AND #5 AND #6 |

PubMed search strategy as an example.
