# Supplementary material for: Effects of aerobic and resistance training on walking and balance abilities in older adults with Parkinson’s disease: A systematic review and meta-analysis
Source: PLoS One. 2025 Jan 9;20(1):e0314539. doi: 10.1371/journal.pone.0314539 (PMC11717240; doi:10.1371/journal.pone.0314539)
Supplement: S4 File — (DOCX) [file pone.0314539.s004.docx]

**S4 File. Included Studies**

| **No.** | **Included studies** | **Age** | **Participants (M/F)** | **Sample Size (T/C)** | **H&Y stage;** **Disease**  **duration** | **Intervention** | | | **Control**  **Group** | **Outcome Measure** |
| --- | --- | --- | --- | --- | --- | --- | --- | --- | --- | --- |
|  |  |  |  |  |  | **Training movement** | **Intensity** | **Frequency;**  **Duration** |  |  |
| 1 | Batista 2016 | T: 64.1 ± 9.1  C: 64.2 ± 8.3 | 19/7 | 13/13 | 2.5;  9.6±3.9/10.7±6.1  years | RT: Leg press, dorsi pulldown, ankle plantarflexion, chest press, half squat | Maximize training adaptations | 60min/times  2 times/week  12 weeks | No exercising | UPDRS-III;  TUG;  MoCA;  PDQ-39 |
| 2 | Cabrera 2020 | T: 77.22 ± 6.22  C: 75.87 ±1.19 | 25/17 | 22/20 | 2-3;NR | RT: Core stabilization  training | NR | 45 min/times  3 times/week; 8 weeks | Routine exercises | Mini-BESTest |
| 3 | Carvalho 2015 | Average:  61.5 ± 9.9 | 15/7 | AT/C: 5/9  RT/C :  8/9 | 1-3;NR | AT: Treadmill walking 30min  RT: Large muscle groups on machines(2 × 8–12 reps) | 70% MHR  70-80% 1RM | 2 times/week  12 weeks | Physiothe-rapy | 2-MST  10-MWT;BBS; UPDRS-III;  MMSE |
| 4 | Granziera 2021 | Average:  68.5±8.3 | 21/11 | 16/16 | 2-3;NR | AT: Nordic Walking | 60 min/times | 8 weeks | Routine walking | UPDRS-III ;  6WMT; 10mWT;  TUG |
| 5 | Kim 2023 | AT(a): 72  AT(b): 61.5  C: 65 | 15/15 | AT(a)/C: 9/11  AT(b)/C :  10/11 | 1-2; ≤5 years | AT(a):HIIT  AT(b):MICT | 60%MP 50S  50%VO2peak | 40-60 min/times  3 times/week;  24 weeks | Usual care | 6MWT;  30CST |
| 6 | F. Li  2012 | AT: 68 ± 9  ST:69 ±8  C: 69 ± 9 | 122/73 | AT)/C: 65/65  RT/C :  65/65 | 1-4;  8±9 years | AT: Tai Chi  RT: Weighted vests and ankle weights | NR  Weighted vests (1-2% body weight) | 60 min/times  2 times/week;  24 weeks | Stretching  exercise | UPDRS-III ;  TUG;  Gait velocity |
| 7 | Li  2022 | AT(a): 62.7±5.51  AT(b): 61.9±5.64  C: 61.9±6.67 | 58/37 | AT(a)/C: 32/32  AT(b)/C:31/32 | 1-2.5;  5.91±4.01 years | AT(a): Tai Chi  AT(b): Brisk walking | NR | 6 months/12months | No exercise | BBS;  UPDRS-III ;  TUG;  Gait velocity |
| 8 | Linder 2022 | T: 64.9 ± 5.5  C: 64.23 ± 8.4 | 11/17 | 14/14 | 1-3;  NR | AT: Aerobic cycling intervention | 60-80% MHR | 30min/times  5 times/week  8 weeks | No exercising | Gait velocity;  Step length;  Cadence; |
| 9 | Mak 2021 | T: 61.9 ± 6.4  C: 62.7 ± 7.2 | 20/44 | 33/31 | 2-3;  5.8±6.1/ 5.0±4.0 years | AT: Brisk walking and balance exercise | NR | weeks 1–6: once/week, weeks 7–26: once/month  6 months | Upper limb training | MDS-UPDRS  6MWD;  Mini-BESTest;  Gait velocity;  TUG |
| 10 | Rawson 2019 | T: 76.05±21.46  C: 79.47±20.81 | 42/28 | 31/39 | 1-4;  5.6±3.8/ 6.1±4.8  years | AT: Walking on treadmill | Preferred walking speed  . | 60min/times  12 weeks | Tango | MDS-UPDRS-III;  6MWD;  Mini-BESTest;  Gait velocity;  PDQ-39 |
| 11 | Schlenstedt 2015 | T: 75.7 ± 5.5  C: 75.7 ±7.2 | 21/11 | 17/15 | 2.5-3.0;  10.1±6.0/9.3±7.9 years | RT: Squats, knee extensions, toe/calf raises, hip abductions | Moderate level | 60min/times  2times/week  7 weeks | Balance training | FAB;  TUG;  MDS-UPDRS;  Gait velocity;  Step length |
| 12 | Shulman2013 | T: 65.6 ± 11.3  C: 69.6 ± 10.7 | 23/22 | 32/13 | 2-3;  6.2±3.8 years | AT: Higher-Intensity Treadmill Training | 70-80% MHR | 3times/week  12 weeks | Lower-Intensity Treadmill Training | 6MWD;  VO_2_ |
| 13 | Silva 2019 | T: 63.12 ±13.61  C: 64.23 ±13.45 | 11/14 | 14/11 | 1-3;  NR | AT: Dual-task  aquatic exercise program | NR | 40min/times  2times/week  12weeks | Routine exercises | BBS;  TUG;  DGI |
| 14 | Ortiz 2018 | T:74.20 ± 5.80  C: 75.4 ± 6.5 | 31/15 | 23/23 | 1-3; 4.0±2.1/4.3±1.9 years | RT: Group resistance training program | 2.7 kg Resistance | 60min/times  2times/week  8weeks | Routine exercises | Mini-BESTest; Piper Fatigue; Scale;  DG |
| 15 | Vieira 2020 | T: 55 ± 7.41  C: 50 ± 8.89 | 30/10 | 25/15 | 1-3; 5.7±0.8/7.2±1.9 years | RT: Progressive resistance training | NR | 50-60min/times  2 times/week  9weeks | No exercising | 10TMW;  TUG;  30CST |

**M, male; F, female; AT, aerobic training; RT, resistance training; PDQ-39, Parkinson’s Disease Questionnaire 39; UPDRS-M, motor section of the Unified Parkinson’s Disease Rating Scale; MoCA, Montreal Cognitive Assessment; TUG, Timed Up & Go Test; FOG-Q, Freezing of Gait Questionnaire; 1RM，one repetition maximum；MHR，maximum heart rate；NR, not recorded; 2-MST, 2-Minute Step Test ; 8-FT, 8-Foot Up and Go Test; 10-MWT, 10-Meter Walk Test; Berg, Berg Balance Scale；Mini-BESTest，Mini-Balance Evaluation Systems Test; ABC score, Activities-specific Balance Confidence Scale; FAB, Fullerton Advanced Balance scale ; HIIT ,high-intensity interval training ; MICT ,moderate-intensity continuous training; MP, maximum aerobic power; 30CST, 30s chair-stand test ;BBS, Berg Balance Scale.**
