# Supplementary material for: Effects of aerobic and resistance training on walking and balance abilities in older adults with Parkinson’s disease: A systematic review and meta-analysis
Source: PLoS One. 2025 Jan 9;20(1):e0314539. doi: 10.1371/journal.pone.0314539 (PMC11717240; doi:10.1371/journal.pone.0314539)
Supplement: S5 File — (DOCX) [file pone.0314539.s005.docx]

**S5 File. Data extracted from the primary research sources**

| **Name of Data extractors** | **Date of**  **Data extraction** | **Included studies** | **Age** | **Sample (T/C)** | **Outcomes** | | | |
| --- | --- | --- | --- | --- | --- | --- | --- | --- |
|  |  |  |  |  | **UPDRS-III scores**  ( Mean ± SD) | **Gait velocity**  ( Mean ± SD) | **Mini-BESTest scores**  ( Mean ± SD) | **TUG**  ( Mean ± SD) |
| HS Song and J LI | 26nd July, 2024 | Batista 2016 | T: 64.1 ± 9.1  C: 64.2 ± 8.3 | 13/13 | RT:41.2 ± 8.4  C: 45.3 ± 6.9 | NA | NA | RT: 9.2 ± 2.3  C: 8.3 ± 1.9 |
| HS Song and J LI | 26nd  July, 2024 | Cabrera 2020 | T: 77.22 ± 6.22  C: 75.87 ±1.19 | 22/20 | NA | NA | RT:22.75 ± 2.86  C: 19.1 ± 3.92 | NA |
| HS Song and J LI | 26nd  July, 2024 | Carvalho 2015 | Average:  61.5 ± 9.9 | AT/C: 5/9  RT/C :8/9 | AT: 20.2 ± 5.5  RT:30.5 ± 8.3  C: 33.9 ± 17 | NA | NA | NA |
| HS Song and J LI | 26nd  July, 2024 | Granziera 2021 | Average:  68.5±8.3 | 16/16 | NA | NA | NA | AT:12.3 ± 2.7  C: 8.9 ± 0.6 |
| HS Song and J LI | 26nd  July, 2024 | Kim 2023 | AT(a): 72  AT(b): 61.5  C: 65 | AT(a)/C: 9/11  AT(b)/C :10/11 | NA | NA | NA | AT(a): 7.4 ± 0.4  AT(b):7.9 ± 0.5  C: 10 ± 0.7 |
| HS Song and J LI | 26nd  July, 2024 | F. Li  2012 | AT: 68 ± 9  RT:69 ±8  C: 69 ± 9 | AT/C: 65/65  RT/C :65/65 | AT: 8.86 ± 4.12  RT:10.25 ± 4.83  C: 13.66 ± 7.54 | AT: 1.2 ± 0.22  RT:1.19 ± 0.24  C: 1.06 ± 0.2 | NA | AT: 7.55 ± 2.69  RT:7.95 ± 2.6  C: 8.67 ± 3.45 |
| HS Song and J LI | 26nd  July, 2024 | Li  2022 | AT(a):  62.7±5.51  AT(b): 61.9±5.64  C: 61.9±6.67 | AT(a)/C: 32/32  AT(b)/C:31/32 | NA | AT(a): 0.95 ± 0.23  AT(b):1.01 ± 0.14  C: 1.1 ± 0.06 | NA | AT(a): 10.45 ± 3.18  AT(b):9.31 ± 1  C: 10.3 ± 1 |
| HS Song and J LI | 26nd  July, 2024 | Linder 2022 | T: 64.9 ± 5.5  C: 64.23 ± 8.4 | 14/14 | NA | AT: 1 ± 0.23  C: 0.8 ± 0.29 | NA | NA |
| HS Song and J LI | 26nd  July, 2024 | Mak 2021 | T: 61.9 ± 6.4  C: 62.7 ± 7.2 | 33/31 | AT: 23.7 ± 6.9  C: 27.3 ± 8.2 | AT: 1.62 ± 0.2  C: 1.46 ± 0.25 | AT: 24.2 ± 2.8  C: 20.5 ± 4.2 | AT: 9.8 ± 3.4  C: 10 ± 1.5 |
| HS Song and J LI | 26nd  July, 2024 | Rawson 2019 | T: 76.05±21.46  C: 79.47±20.81 | 31/39 | AT: 109.57 ± 4.65  C: 103.36 ± 4.32 | NA. | AT: 18.97 ± 0.93  C: 19.58 ± 0.62 | NA |
| HS Song and J LI | 26nd  July, 2024 | Schlenstedt 2015 | T: 75.7 ± 5.5  C: 75.7 ±7.2 | 17/15 | RT:19.9 ± 8.9  C: 19.4 ± 6.7 | RT:30.5 ± 8.3  C: 33.9 ± 17 | NA | RT: 9.5 ± 2.4  C : 9 ± 1.8 |
| HS Song and J LI | 26nd  July, 2024 | Shulman2013 | T: 65.6 ± 11.3  C: 69.6 ± 10.7 | 32/13 | AT: 7.4 ± 0.4  C: 10 ± 0.7 | NA | NA | NA |
| HS Song and J LI | 26nd  July, 2024 | Silva 2019 | T: 63.12 ±13.61  C: 64.23 ±13.45 | 14/11 | NA | NA | NA | AT: 13.31 ± 2.83  C: 16.68 ± 3.42 |
| HS Song and J LI | 26nd  July, 2024 | Ortiz 2018 | T:74.20 ± 5.80  C: 75.4 ± 6.5 | 23/23 | NA | NA | RT:19.4 ± 3.6  C: 17.2 ± 3.1 | NA |
| HS Song and J LI | 26nd  July, 2024 | Vieira 2020 | T: 55 ± 7.41  C: 50 ± 8.89 | 25/15 | NA | RT: 2 ± 0.3  C: 1.7 ± 0.3 | NA | NA |

Note: AT, Aerobic training; RT, resistance training;C, Control Group; NA, Not available.
