# Supplementary material for: Effects of aerobic and resistance training on walking and balance abilities in older adults with Parkinson’s disease: A systematic review and meta-analysis
Source: PLoS One. 2025 Jan 9;20(1):e0314539. doi: 10.1371/journal.pone.0314539 (PMC11717240; doi:10.1371/journal.pone.0314539)
Supplement: S6 File — (DOCX) [file pone.0314539.s006.docx]

**S6 File. Quality evaluation**

| **Study** | **Each Question** | | | | | | | | | | | **Total score** |
| --- | --- | --- | --- | --- | --- | --- | --- | --- | --- | --- | --- | --- |
|  | 1 | 2 | 3 | 4 | 5 | 6 | 7 | 8 | 9 | 10 | 11 |  |
| Carvalho 2015 | Y | N | Y | Y | N | N | N | Y | Y | Y | Y | 7 |
| Cabrera 2020 | Y | Y | Y | Y | N | N | Y | Y | Y | Y | Y | 9 |
| Granziera 2021 | Y | Y | Y | Y | N | N | Y | Y | Y | Y | Y | 9 |
| Kim 2023 | Y | N | N | Y | N | N | N | Y | Y | Y | Y | 6 |
| F.Li 2012 | Y | Y | Y | Y | N | N | N | Y | Y | Y | Y | 8 |
| Li 2022 | Y | N | Y | Y | Y | N | Y | Y | Y | Y | Y | 9 |
| Mak 2021 | Y | Y | NA | Y | Y | N | N | Y | Y | Y | Y | 8 |
| Rawson 2019 | Y | Y | N | Y | N | N | N | Y | Y | NA | Y | 6 |
| Schlenstedt, 2015 | Y | Y | N | Y | N | NA | N | Y | Y | Y | Y | 7 |
| Shulman, 2013 | Y | N | N | Y | N | N | N | Y | Y | Y | Y | 6 |
| Batista 2016 | Y | Y | N | Y | Y | N | N | Y | Y | Y | Y | 8 |
| Vieira 2020 | Y | Y | Y | Y | N | NA | Y | Y | Y | Y | Y | 9 |
| Ortiz 2018 | Y | Y | Y | Y | N | N | N | Y | Y | Y | Y | 8 |
| Silva 2019 | Y | NA | N | Y | N | N | N | Y | Y | NA | Y | 5 |
| Linder 2022 | Y | Y | N | Y | N | N | N | Y | Y | Y | Y | 7 |

**Key: Y= Yes; N= Not, NA=Not appropriate**

**Question codes:**

**1. Was eligibility criteria specified?**

**2. Were all subjects randomly allocated?**

**3. Were allocations concealed?**

**4. Were the groups similar at baseline?**

**5. Was there blinding of all participants?**

**6. Was there blinding of all therapists?**

**7. Was there blinding of all assessors?**

**8. Was there measures of at least one key outcome for more than 85% of the subjects initially allocated to groups?**

**9. Did all subjects for whom outcome measures were available receive the treatment or control condition as allocated or, where this was not the case, data for at least one key outcome was analyzed by “intention to treat”**

**10. Were the results of between group statistical comparisons reported for at least one key outcome?**

**11. Did the study have both point measures and measures of variability for at least one key outcome.**
